# Brain pyrimidine nucleotide synthesis and Alzheimer disease

**DOI:** 10.18632/aging.102328

**Published:** 2019-09-27

**Authors:** Alba Pesini, Eldris Iglesias, M.Pilar Bayona-Bafaluy, Nuria Garrido-Pérez, Patricia Meade, Paula Gaudó, Irene Jiménez-Salvador, Pol Andrés-Benito, Julio Montoya, Isidro Ferrer, Pedro Pesini, Eduardo Ruiz-Pesini

**Affiliations:** 1 Departamento de Bioquímica, Biología Molecular y Celular, Universidad de Zaragoza, Zaragoza, Spain; 2 Instituto de Investigación Sanitaria de Aragón (IIS Aragón), Zaragoza, Spain; 3 Centro de Investigaciones Biomédicas en Red de Enfermedades Raras (CIBERER), Madrid, Spain; 4 Departamento de Patología y Terapéutica Experimental, Universidad de Barcelona, Hospitalet de Llobregat, Barcelona, Spain; 5 Centro de Investigaciones Biomédicas en Red de Enfermedades Neurodegenerativas (CIBERNED), Madrid, Spain; 6 Instituto de Investigación Biomédica de Bellvitge (IDIBELL), Hospitalet de Llobregat, Barcelona, Spain; 7 Servicio de Anatomía Patológica, Hospital Universitario de Bellvitge, Hospitalet de Llobregat, Barcelona, Spain; 8 Instituto de Neurociencias, Universidad de Barcelona, Barcelona, Spain; 9 Araclon Biotech, Zaragoza, Spain; 10 Fundación ARAID, Zaragoza, Spain

**Keywords:** Alzheimer disease, brain, pyrimidine biosynthesis *de novo*, pyrimidine salvage pathway, oxidative phosphorylation

## Abstract

Many patients suffering late-onset Alzheimer disease show a deficit in respiratory complex IV activity. The *de novo* pyrimidine biosynthesis pathway connects with the mitochondrial respiratory chain upstream from respiratory complex IV. We hypothesized that these patients would have decreased pyrimidine nucleotide levels. Then, different cell processes for which these compounds are essential, such as neuronal membrane generation and maintenance and synapses production, would be compromised. Using a cell model, we show that inhibiting oxidative phosphorylation function reduces neuronal differentiation. Linking these processes to pyrimidine nucleotides, uridine treatment recovers neuronal differentiation. To unmask the importance of these pathways in Alzheimer disease, we firstly confirm the existence of the *de novo* pyrimidine biosynthesis pathway in adult human brain. Then, we report altered mRNA levels for genes from both *de novo* pyrimidine biosynthesis and pyrimidine salvage pathways in brain from patients with Alzheimer disease. Thus, uridine supplementation might be used as a therapy for those Alzheimer disease patients with low respiratory complex IV activity.

## INTRODUCTION

Late-onset Alzheimer disease (AD) is a chronic and progressive neurodegenerative disorder clinically characterized by memory loss and cognitive decline. Representative histopathological findings in AD brain include extracellular amyloid-*β* (A*β*) plaques, intracellular neurofibrillary tangles composed of hyperphosphorylated tau, and a continuous loss of neurons. According to the ‘amyloid cascade hypothesis’, A*β* deposition is the cause of AD [1]. However, in the ‘mitochondrial cascade hypothesis’, the origin of AD is a defect in the oxidative phosphorylation (OXPHOS) system [2]. Interestingly, bioenergetics and A*β* appear to be closely related. Thus, A*β* can reduce OXPHOS function and OXPHOS deficiency can increase A*β* production [3].

**Figure 1:**
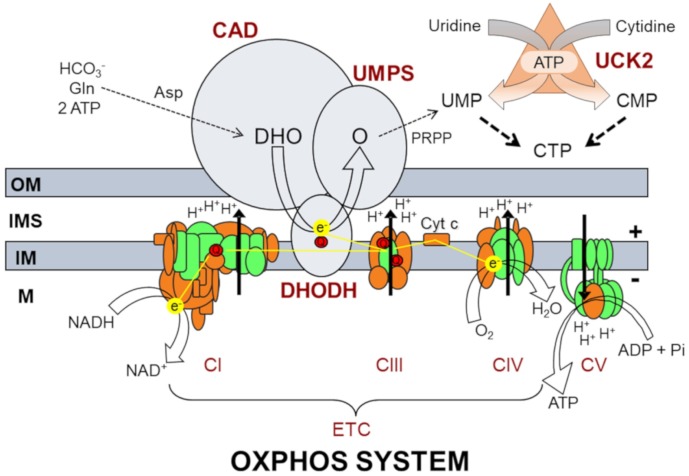
**Oxidative phosphorylation system (OXPHOS) and biochemical pathways for pyrimidine nucleotide synthesis.** OM, IMS, IM, and M code for mitochondrial outer membrane, intermembrane space, mitochondrial inner membrane, and mitochondrial matrix, respectively; ETC, electron transport chain; CI, CIII, CIV, CV and Cyt c code for respiratory complexes I, III, IV, ATP synthase and cytochrome c, respectively; Q, coenzyme Q_10_; NADH and NAD^+^, reduced and oxidized forms of nicotinamide adenine dinucleotide; H^+^, protons; e^-^, electrons; ADP, ATP and Pi, adenosine diphosphate, adenosine triphosphate, and inorganic phosphate; H_2_O, water; O_2_, oxygen; CAD, carbamoyl phosphate synthetase, aspartate transcarbamylase, dihydroorotase; DHODH, dihydroorotate dehydrogenase; UMPS, uridine monophosphate synthase; HCO_3_^-^, bicarbonate; Gln, glutamine; Asp, aspartate; DHO, dihydroorotate; O, orotate; PRPP, phosphoribosyl 5’-pyrophosphate; UMP, uridine monophosphate; CMP, cytidine monophosphate; CTP, cytidine triphosphate; UCK2, uridine cytidine kinase 2.

OXPHOS is much more than an energy-generating cellular pathway [4]. OXPHOS dysfunction can affect many biochemical routes, among them the *de novo* pyrimidine biosynthesis. This process requires three proteins: CAD, named after its three enzyme activities, carbamoyl-phosphate synthetase, aspartate transcarbamylase and dihydroorotase; DHODH, dihydroorotate dehydrogenase; and UMPS, which enzyme activities are orotate phosphoribosyltransferase and orotidine-5′-phosphate decarboxylase (Figure 1). DHODH is located in the mitochondrial inner membrane and directly transfers electrons to the OXPHOS electron transport chain (ETC) via coenzyme Q_10_ [5]. A reduction of electron transport downstream coenzyme Q_10_ would slow down DHODH activity and the *de novo* pyrimidine biosynthesis.

Pyrimidine nucleotides are required for the synthesis of nucleic acids, carbohydrates, and many membrane components, such as glycoproteins, glycolipids, and phospholipids. As proliferating cells need to duplicate their genomes and other cell components like biological membranes, they depend on high concentrations of pyrimidine nucleotides. Then, the *de novo* pyrimidine biosynthesis pathway is indispensable for these cells. However, differentiated cells do not divide themselves or replicate their genomes. Hence, it is generally considered that the activity of *de novo* route is low in these cells and they satisfy their need for pyrimidine nucleotides through the salvage pathway [5]. In this process, the uridine-cytidine kinase 2 (UCK2) phosphorylates uridine and cytidine nucleosides to produce UMP and CMP [6].

Neurons are post-mitotic, differentiated cells. Neuronal differentiation includes the formation of axons and dendrites and the maintenance of the neuron’s vastly expanded surface. These events require a continuous membrane synthesis, even in adult’s post-mitotic neurons [7]. Moreover, it has been observed that highly elaborate axonal arborization of neurons greatly increases their baseline energy demands, rendering neurons more vulnerable to perturbations of mitochondrial function pathways [8]. We had previously hypothesized that an OXPHOS dysfunction in AD patients can secondarily affect *de novo* pyrimidine biosynthesis and the plasma membrane remodeling [9]. This might explain the alterations in composition and structure of neuronal membranes linked to loss of synapsis that precede neuronal loss in AD patients [10]. Therefore, pyrimidine nucleoside supplementation could be used as a therapeutic agent in AD.

## RESULTS AND DISCUSSION

### OXPHOS dysfunction impairs neuronal differentiation through altered *de novo* pyrimidine biosynthesis

### Expression of enzymes from pathways for pyrimidine nucleotide synthesis in neurons differentiated from neuroblastoma SH-SY5Y cells

**Figure 2:**
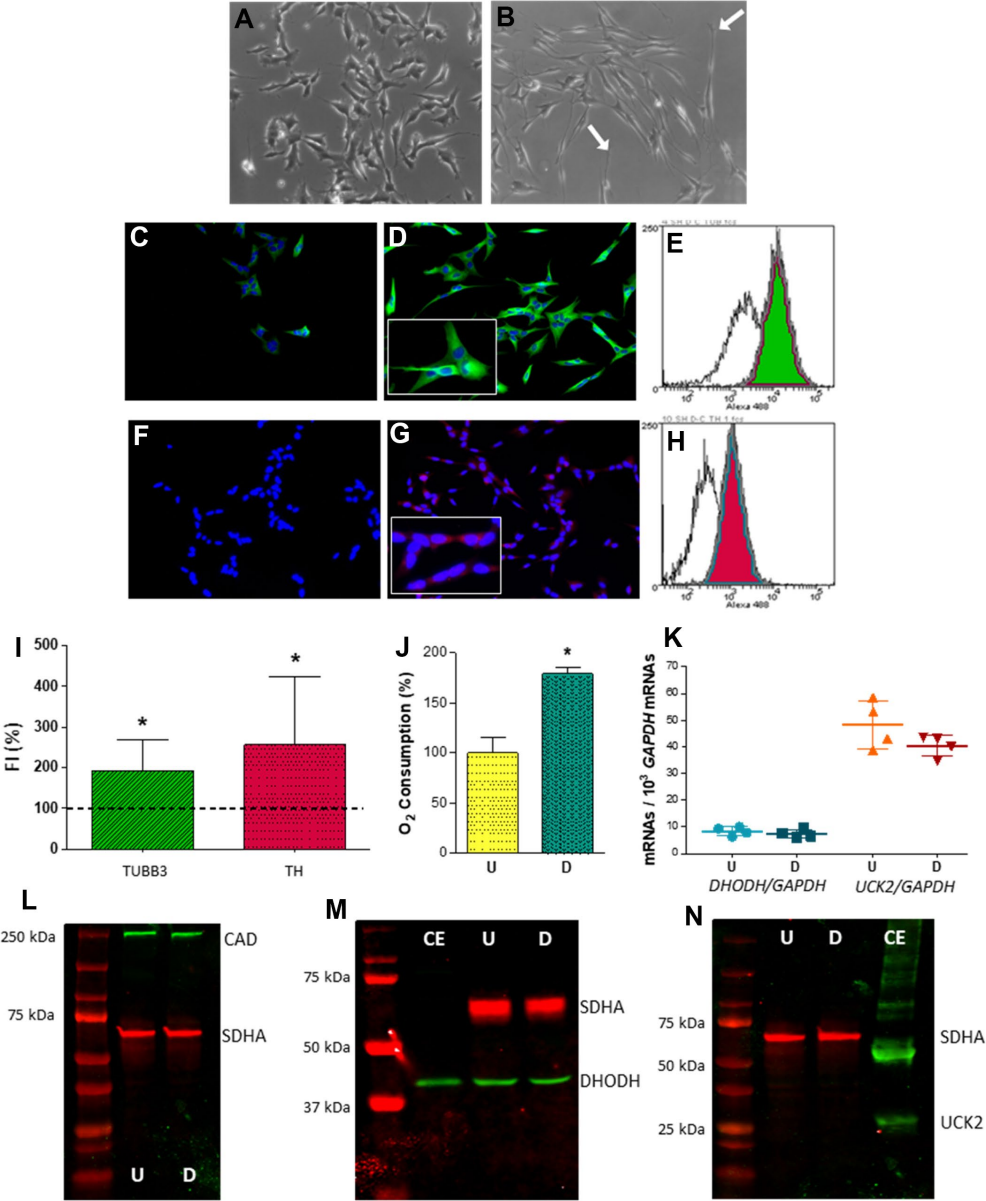
**Expression of selected genes from pyrimidine nucleotide synthesis pathways in human neuroblastoma SH-SY5Y cells.** (**A**, **B**) Representative optic microscopy images of (**A**) undifferentiated and (**B**) neuron-differentiated SH-SY5Y cells. White arrows point to neurites. (**C**, **D**) Representative immunofluorescence microscopy images of anti-TUBB3 stained (**C**) undifferentiated and (**D**) neuron-differentiated SH-SY5Y cells. Inset: enlarged figure detail. (**E**) Representative image of a flow cytometry histogram of anti-TUBB3 stained undifferentiated (white) and neuron-differentiated (green) cells. (**F**, **G**) Representative immunofluorescence microscopy images of anti-TH stained (**F**) undifferentiated and (**G**) neuron-differentiated SH-SY5Y cells. Inset: enlarged figure detail. (**H**) Representative image of a flow cytometry histogram of anti-TH stained undifferentiated (white) and neuron-differentiated (red) cells. (**I**) Graph showing the change of fluorescence intensity (FI) in TUBB3 and TH levels after neuronal differentiation. Dashed line (100 %) represents TUBB3 or TH mean values of undifferentiated cells. Bars indicate mean values and standard deviations in differentiated cells. N = 11. *: p < 0.05 (versus undifferentiated cells). (**J**) Oxygen consumption of (U) undifferentiated and (**D**) neuron-differentiated cells. N = 3. *: p < 0.05 (versus undifferentiated cells). (**K**) *DHODH* and *UCK2* mRNA levels in (U) undifferentiated and (**D**) neuron-differentiated cells. Points represent individual samples and horizontal lines indicate mean ± standard deviation values. N = 4. (**L**–**N**) Representative images of western blots for (**L**) CAD (N = 2), (**M**) DHODH (N = 3) and (**N**) UCK2 (N = 2) proteins. CE, commercial enzyme; SDHA, succinate dehydrogenase subunit A. 70 μg of cell protein was used in these western blots (**L**–**N**).

To check a potential effect of the OXPHOS dysfunction on neuronal pyrimidine nucleotide synthesis, we used the human neuroblastoma SH-SY5Y cell line as a model. This cell line has frequently been used to study AD-related issues. In fact, considering ‘SH-SY5Y’ and ‘Alzheimer’s’ terms, more than 1,400 publications appear in PubMed database. These cells can be differentiated into dopaminergic neurons. They express βIII-tubulin (TUBB3), a neuronal marker, and tyrosine hydroxylase (TH), a dopaminergic marker (Figure 2A–2I). Neuronal differentiation is also accompanied by an increase in oxygen consumption (Figure 2J).

Proliferating cells express both *DHODH* and *UCK2* mRNAs (Figure 2K). Levels are very similar to those reported in the human protein atlas webpage (https://www.proteinatlas.org/). *DHODH* mRNA levels were approximately five times lower than those for *UCK2* mRNA. Neuronal differentiation did not modify mRNA values. The UCK2 protein was not detected in proliferating cells, although CAD and DHODH proteins were found (Figure 2L–2N). As UCK2 was previously reported in proliferating SH-SY5Y cells [11], we ruled out a general lack of pyrimidine salvage pathway in these cells. Perhaps the anti-UCK2 antibody was not sensitive enough. In fact, we needed 10 μg of a commercial enzyme to obtain a faint band in the Western blot. Additionally, we confirmed the presence of CAD and DHODH, but no UCK2, in these post-mitotic cells, i.e. neuron-differentiated SH-SY5Y cells (Figure 2L–2N).

### Neuronal differentiation after DHODH inhibition

**Figure 3:**
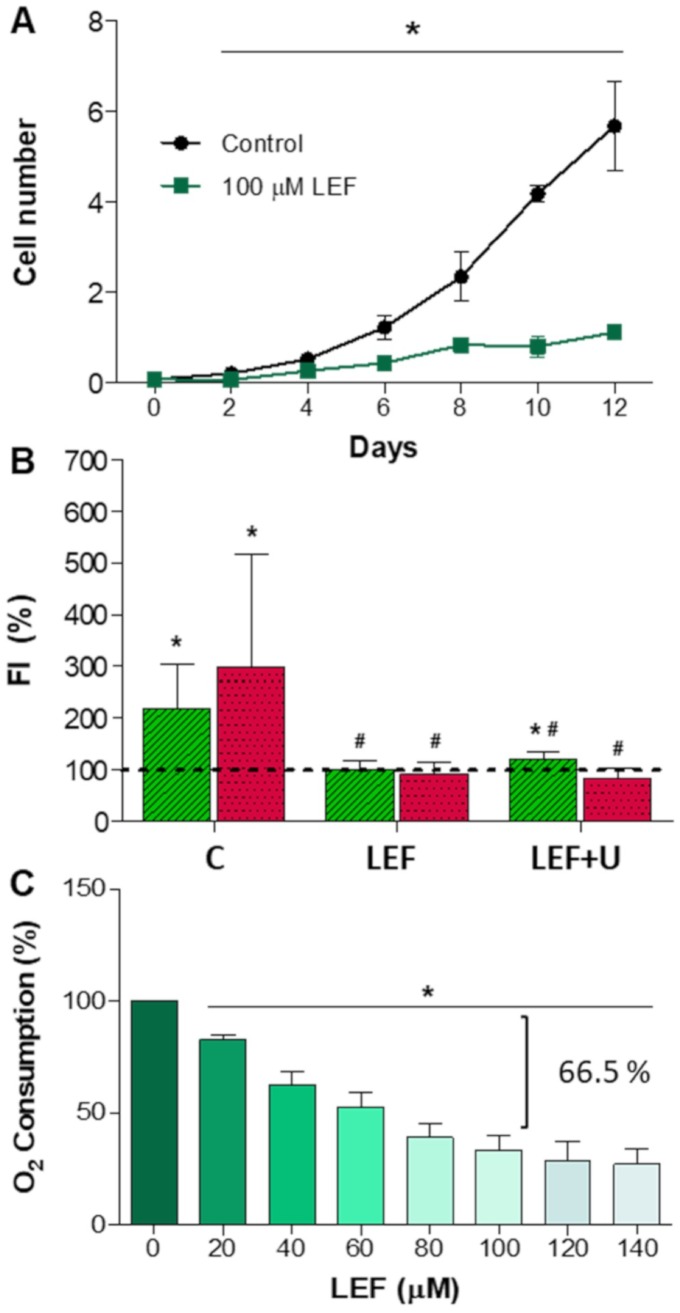
**Leflunomide effect on neuroblastoma SH-SY5Y cells.** N ≥ 3. (**A**) Cell proliferation (in million cells). *: p < 0.05 versus untreated cells (control). (**B**) TUBB3 (green) and TH (red) fluorescence intensity (FI) mean and standard deviation values in neuron-differentiated cells. Dashed line (100 %) represents TUBB3 or TH mean values of undifferentiated cells. C: untreated cells; LEF: leflunomide-treated cells; LEF+U, leflunomide plus uridine (200 μM)-treated cells; *: p ≤ 0.0021, versus undifferentiated cells; ^#^: p ≤ 0.0055, versus untreated neuron-differentiated cells. (**C**) Oxygen consumption in proliferating SH-SY5Y cells. *: p < 0.05, versus untreated cells.

As previously commented upon, *de novo* pyrimidine biosynthesis is relevant for proliferating cells. Orally administered leflunomide is almost completely converted into its active metabolite teriflunomide and, inhibiting DHODH, it decreases pyrimidine nucleotide availability and cell proliferation. Because of this, it has been used for treatment of rheumatoid arthritis and it is also a potent anticancer drug [12]. During patient treatment, teriflunomide steady-state plasma concentrations of 200 μM can be reached [13]. We observed that leflunomide 100 μM largely reduces cell proliferation of human neuroblastoma SH-SY5Y cells (Figure 3A).

According to our hypothesis, *de novo* pyrimidine biosynthesis pathway is also important for neurons. To revel its role in these cells, we studied the leflunomide effect on neuronal differentiation. Leflunomide 100 μM prevents an increase in TUBB3 and TH levels (Figure 3B). It has been shown that uridine can completely overcome growth inhibition by leflunomide [14]. However, uridine 200 or 800 μM were not able to prevent the effect of leflunomide on neuronal differentiation (Figure 3B).

The additional inhibition of other cell functions by leflunomide, besides its effect on the *de novo* pyrimidine biosynthesis pathway, might be the reason why uridine has no effect in the maintaining neuronal differentiation [12]. In human cells, leflunomide reduces the mitochondrial inner membrane potential [15]. Mitochondrial inner membrane potential is generated by the electron flow through the respiratory chain, and this electron flow is associated with oxygen consumption. To determine the leflunomide effect on oxygen consumption of neuroblastoma SH-SY5Y cells, we tested different leflunomide concentrations. Leflunomide 100 μM reduces oxygen consumption to a residual 33.5 % (Figure 3C). Supporting our results, it has been reported that leflunomide, or its metabolite teriflunomide, decreased oxygen consumption in mouse and human cells [13, 16–20]. These results suggest that an important part of oxygen consumption and energy production in proliferating cells is due to pyrimidine biosynthesis. *DHODH* knockdown diminished mitochondrial ATP production and inner membrane potential in mouse and human cells [21, 22]. Leflunomide causes mitochondrial proliferation in human cells [15], suggesting a compensatory response. Moreover, externally added uridine to the culture media did not improve the mitochondrial inner membrane potential and did not prevent mitochondrial proliferation, despite that uridine normalizes the cell cycle progression [15]. Uridine 200 μM do not recover oxygen consumption of leflunomide-treated cells (Supplementary Figure 1). Similarly, it was reported that uridine had no protective effect against tamoxifen-induced impairment to mitochondrial respiration [23].

### Neuronal differentiation after OXPHOS inhibition

**Figure 4:**
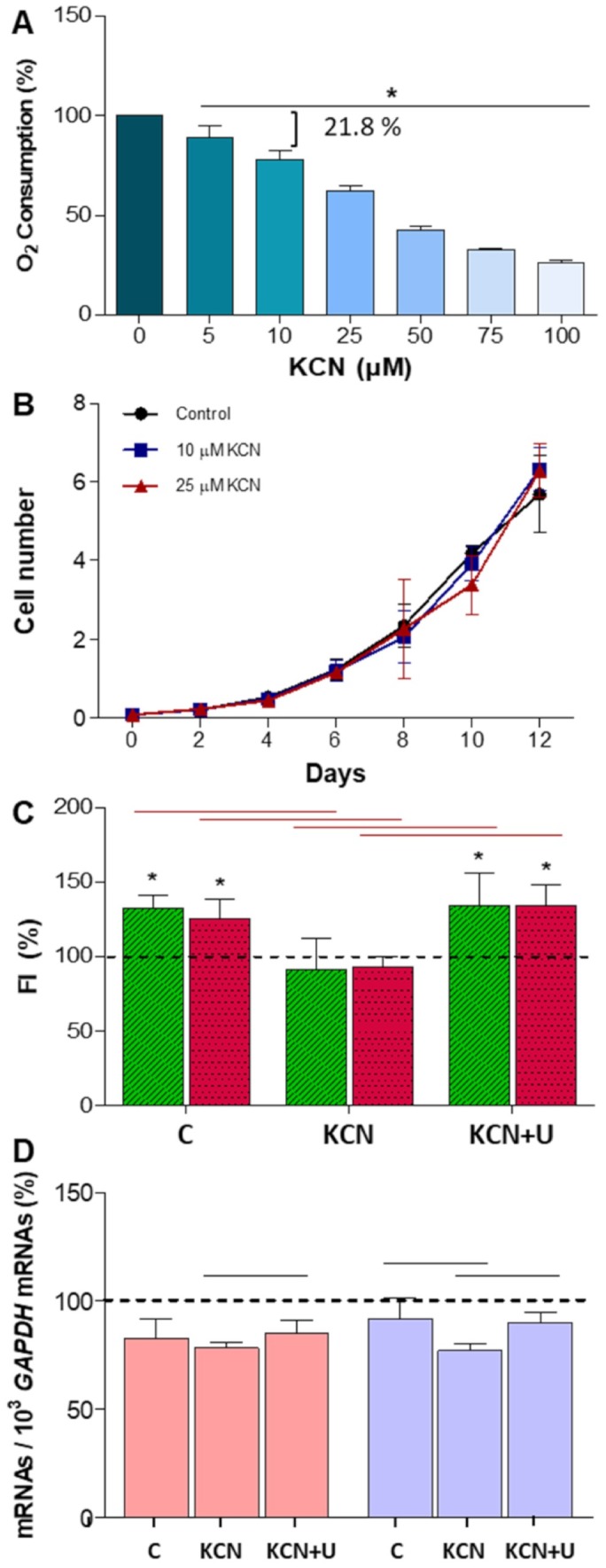
**Potassium cyanide (KCN) effect on neuroblastoma SH-SY5Y cells.** N ≥ 3. (**A**) Oxygen consumption in proliferating SH-SY5Y cells at increasing KCN concentrations. *: p < 0.05, versus untreated cells. (**B**) Cell proliferation (in million cells) without KCN (control) and at 10 or 25 μM KCN. (**C**) TUBB3 (green) and TH (red) fluorescence intensity (FI) mean and standard deviation values in neuron-differentiated cells. Dashed line (100 %) represents TUBB3 or TH mean values of undifferentiated cells. *: p < 0.05, versus undifferentiated cells. Horizontal red lines indicate p values ≤ 0.0004 between control untreated neuron-differentiated cells (**C**), KCN-treated neuron-differentiated cells (KCN) and KCN plus uridine-treated neuron-differentiated cells (KCN+U), as indicated. (**D**) *DHODH* (pink) and *UCK2* (purple) mRNA mean and standard deviation values in C, KCN and KCN+U neuron-differentiated cells. Dashed line (100 %) represents *DHODH* and *UCK2* mRNA mean values of undifferentiated cells. Black horizontal lines indicate p values < 0.05 between groups as indicated.

The OXPHOS dysfunction due to leflunomide might be responsible for the reduced neuronal differentiation of neuroblastoma SH-SY5Y cells. Because leflunomide also acts on other cell targets [12], we selected the CIV inhibitor potassium cyanide (KCN) that presumably does not act on these other leflunomide targets. KCN 10 μM or 25 μM decrease oxygen consumption in neuroblastoma SH-SY5Y cells to a residual 78 and 62 %, respectively (Figure 4A). It had been previously shown that KCN abolished DHO-induced oxygen consumption in mouse cells and mitochondria from different rat tissues [16, 24], confirming that OXPHOS CIV inhibition affected the *de novo* pyrimidine biosynthesis pathway. Moreover, in the human neuroblastoma SH-SY5Y cell line, KCN 10 μM and 25 μM do not affect cell proliferation (Figure 4B).

Neuroblastoma SH-SY5Y cells were cultured with KCN 10 μM to analyze the effect of OXPHOS dysfunction on neuronal differentiation. These cells do not show increased TUBB3 and TH levels (Figure 4C). Supporting our results, it was previously reported that KCN significantly inhibited the dopaminergic neuronal differentiation of neural progenitor cells, derived from human induced pluripotent stem cells [22]. Hence, this compound, by reducing respiratory chain activity, prevents dopaminergic neuronal differentiation.

In MDA231 human breast cancer cells, it has been shown that *de novo* pyrimidine synthesis pathway is depressed under low energy conditions, supposedly to reduce ATP-consumption [25]. To determine if that occurs in differentiated cells, we measured *DHODH* and *UCK2* mRNA levels in KCN-treated SH-SY5Y cells after their differentiation to neurons. Although the *de novo* pathway requires more ATP than the salvage one, *DHODH* mRNA expression level did not change, but *UCK2* mRNA levels significantly decreased (Figure 4D). It is worth noting that a high percentage of OXPHOS oxygen consumption is due to electrons supplied to the ETC through the DHODH enzyme. Then, *de novo* pyrimidine biosynthesis is required not only for pyrimidine synthesis and energy production but also for other OXPHOS-related cell functions, such as the adjustment of the levels of signaling molecules (ATP, calcium, NAD^+^ and reactive oxygen species) to modulate different cell pathways [4]. This fact might explain why the *de novo* pathway is not down-regulated in neuron-differentiated cells under low energy conditions, whereas the salvage pathway only involved in pyrimidine synthesis, is reduced.

### Uridine recovers neuronal differentiation of OXPHOS dysfunctional cells

It was previously reported that uridine protected AD fibroblasts and normal human neuronal progenitor cells against azide toxicity [26, 27], another CIV inhibitor. Supplementation of uridine to aged rats increased brain neurofilament levels [28]. Besides, a uridine effect on neuronal differentiation was reported. Thus, in rat PC12 pheochromocytoma cells and mouse N2a and human LAN-5 and SH-SY5Y neuroblastoma cells, exogenous uridine enhanced cell differentiation as evidenced by increased number of neurite-bearing cells, neurites per cell, neurite branching, neurite length, and neurite neurofilament levels [27, 29–31].

We observed that uridine 200 μM was able to restore *UCK2* mRNA levels in KCN-treated neuroblastoma SH-SY5Y cells (Figure 4D). Perhaps an excess of the UCK2 substrate uridine acts as a positive regulator for the expression of *UCK2* mRNA. Uridine completely recovered TUBB3 and TH protein levels in these cells (Figure 4C). Uridine increases the synthesis of UDP-N-Acetylglucosamine (UDP-GlcNAc) [32]. This compound transfers GlcNAc to different proteins, such as transcription factors or histones, which implicates this modification in transcriptional regulation [33, 34]. Maybe, this is the way uridine recovers TUBB3 and TH expression.

All these results indicate that OXPHOS dysfunction affects *de novo* pyrimidine nucleotide biosynthesis pathway and has negative consequences on neurons generated from neuroblastoma SH-SY5Y cells. As previously commented upon, it is considered that the activity of the *de novo* pathway is low in fully differentiated cells, such as mature brain neurons [5], but the cell’s capacity to salvage uridine is limited, and no cell can tolerate complete DHODH inhibition [35]. Moreover, some observations suggest that *de novo* pyrimidine biosynthesis pathway is also important in mature neurons from adult human brain [36].

### The *de novo* pyrimidine biosynthesis pathway is present in adult human brain

### DHODH mRNA

According to RNA-Seq studies reported in the Expression Atlas webpage (https://www.ebi.ac.uk/gxa/ home; accessed April 23, 2018), *CAD*, *DHODH*, and *UMPS* mRNAs are found in brain of primates, rodents, other placental and marsupial mammals, birds, and amphibians (Supplementary Table 1). There are no large differences in the number of transcripts per million for genes from the *de novo* pyrimidine biosynthesis pathway among species. By comparison, *UCK2* mRNA levels, from the pyrimidine salvage pathway, are also very much alike. The presence of these mRNAs in adult rat brain was confirmed by *in situ* hybridization analysis [37]. Neocortex, cerebellar cortex, and hippocampus, which have high neuronal densities, showed high signal intensities.

**Figure 5:**
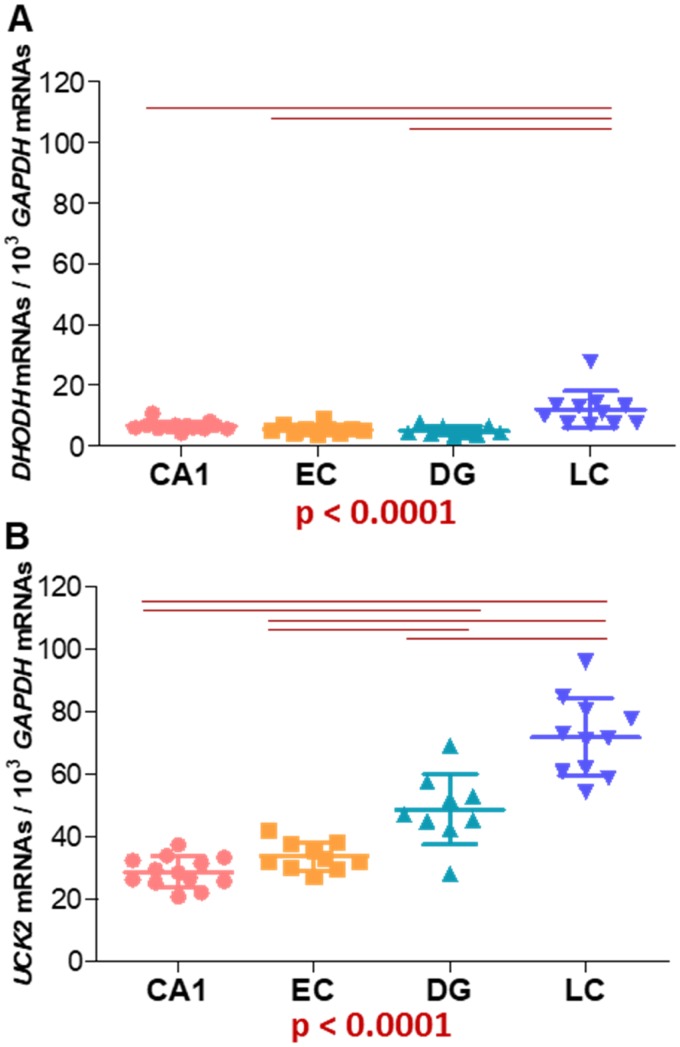
Expression of genes encoding (**A**) *DHODH* and (**B**) *UCK2* proteins from both pyrimidine nucleotide synthesis pathways. *GAPDH* mRNA levels have been used to normalize. CA1: hippocampal cornus ammon 1; EC: entorhinal cortex; DG: dentate gyrus; LC: locus ceruleus. Points represent individual samples and horizontal lines indicate mean ± standard deviation values. P values for Kruskal-Wallis tests are indicated under the graphs. Horizontal red lines indicate between-regions p values (p < 0.0083) fulfilling the post-hoc Bonferroni/Dunn criteria.

Since *CAD*, *DHODH*, and *UMPS* mRNAs are found in brain from different species, expression of these genes may also be in the human brain. We tuned up a RT-qPCR to quantify *DHODH*, normalized by *GAPDH*, mRNA levels in four different regions of adult human brain from clinical and histopathological controls (CHPC) (Supplementary Table 2 and Figure 5A). We also determined *UCK2* mRNA levels (Supplementary Table 2 and Figure 5B). Depending on the brain region, the *DHODH* mRNA amount was 4.3 - 9.7 times lower than that of *UCK2* mRNA. *DHODH* and *UCK2* mRNA levels in the locus ceruleus (LC) were significantly higher than those from the other brain regions. Dentate gyrus (DG) *UCK2* mRNA levels were also significantly different from those of other brain regions (Figure 5A, 5B). A review of the Expression Atlas Webpage (accessed April 23, 2018) showed that *DHODH* and *UCK2*, but also *CAD* and *UMPS*, mRNAs were found in different regions of adult human brain (Supplementary Table 3). Similar mRNA levels were found for different genes, in different brain regions, and in various studies.

### DHODH protein

By using a non-commercial anti-DHODH polyclonal antibody, the immunoblotting of protein from adult rat brain revealed the presence of DHODH in different regions, such as cortex, hippocampus, striatum, cerebellum, brain stem, and spinal cord [38].

**Figure 6:**
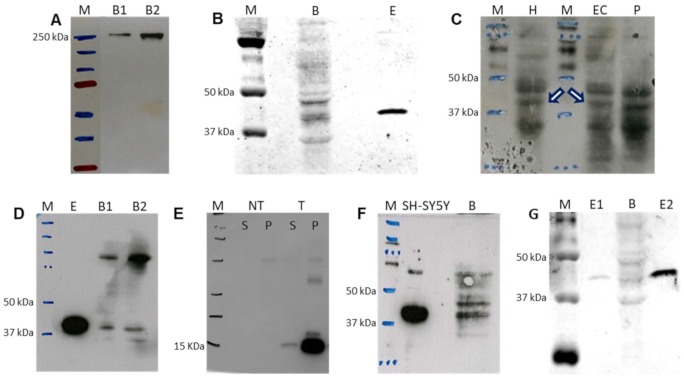
**Western blot detection of selected proteins from the *de novo* pyrimidine biosynthesis pathway in adult human brain.** (**A**) CAD protein in 40 and 80 μg of brain sample, B1 and B2, respectively. (**B**) DHODH protein in 180 μg of brain (**B**) protein using polyclonal antibody. Lane E: commercial DHODH enzyme lacking its 31 first amino acids (250 ng of protein). (**C**) DHODH protein in hippocampus (H, 100 μg of protein), entorhinal cortex (EC, 180 μg of protein) and putamen (P, 180 μg of protein) using polyclonal antibody. White arrows indicate the corresponding band for DHODH. (**D**) DHODH protein using monoclonal antibody for detection of commercial enzyme lacking its first 31 amino acids (E, 250 ng of protein) and in 40 and 80 μg of brain sample, B1 and B2, respectively. (**E**) Fragment of DHODH protein used as immunogen to produce the monoclonal antibody. NT and T homogenates of untransformed bacteria and bacteria transformed with the DHODH fragment sequence, respectively. S and P: supernatant and pellet, respectively. (**F**) DHODH protein in neuroblastoma SH-SY5Y cell line (70 μg of protein) and brain tissue (B, 250 μg of protein) using polyclonal antibody. (**G**) Quantification of brain DHODH protein with the polyclonal antibody in brain (B, 180 μg of protein) by comparison with the commercial enzyme lacking its first 31 amino acids at 0.4 and 4.0 ng (E1 and E2), respectively. M: molecular weight marker.

To confirm the occurrence of enzymes from the *de novo* pyrimidine biosynthesis pathway in adult human brain, we first performed a western blot for CAD. The anti-CAD monoclonal antibody showed a unique band of the expected size (Figure 6A). However, the anti-DHODH polyclonal antibody showed multiple bands (Figure 6B). One of them was located in the expected position for a protein which size is the same of DHODH (43 kDa). Additionally, this signal coincided with the band for a commercial DHODH lacking its first 31 amino acids (ab128451, Abcam, Cambridge, UK), with approximately 40 kDa expected size. The analysis of different brain regions, such as hippocampus, entorhinal cortex (EC), and putamen suggested the presence of DHODH in all of them, but again, multiple bands were observed (Figure 6C). An anti-DHODH monoclonal antibody resulted in a cleaner lane, but still with several bands (Figure 6D). One of them was located in the expected position, coinciding with the site for the commercial DHODH. To produce this monoclonal antibody, a DHODH fragment (amino acid positions 32 -141) was used (ab54621, Abcam, Cambridge, UK). This antibody also recognized the same DHODH fragment generated by us (Figure 6E).

In an attempt to further confirm the presence of this protein in adult human brain, we carried out proteomic analyses of the western blot candidate band. In parallel to the brain sample, we also loaded a sample from neuroblastoma SH-SY5Y cell line, because proliferating cells require a large amount of pyrimidine and are dependent on DHODH, as we have previously shown (Figure 6F). Peptide mass fingerprinting did not show the protein in these samples. However, a parallel reaction monitoring (PRM) proteomics analysis showed 3 DHODH peptides in the western blot band corresponding to neuroblastoma SH-SY5Y cell homogenate (Supplementary Figures 2A and 3), but DHODH could not be confirmed in the band corresponding to brain homogenate, probably due to a high background. Peptide mass fingerprinting and PRM proteomics analyses suggested, but did not confirm, the presence of DHODH in one of 6 spots from a two-dimensional electrophoresis gel (Supplementary Figures 2B and 4). It may be that the brain DHODH quantity was too low. However, a western blot of a brain sample using two different concentrations of a commercial DHODH allowed us to estimate its brain concentration as close to 2.2 ng DHODH/mg brain (Figure 6G). By ELISA, a mean concentration of 128.3 pg DHODH/mg brain was estimated in hippocampus from five control individuals. Surprisingly, the UCK2 mean concentration was much lower, 0.6 pg UCK2/mg brain. These results are in line with those SH-SY5Y cells, in which higher *UCK2* mRNA levels but much lower UCK2 protein concentrations than those for DHODH were observed.

### DHODH activity

**Figure 7:**
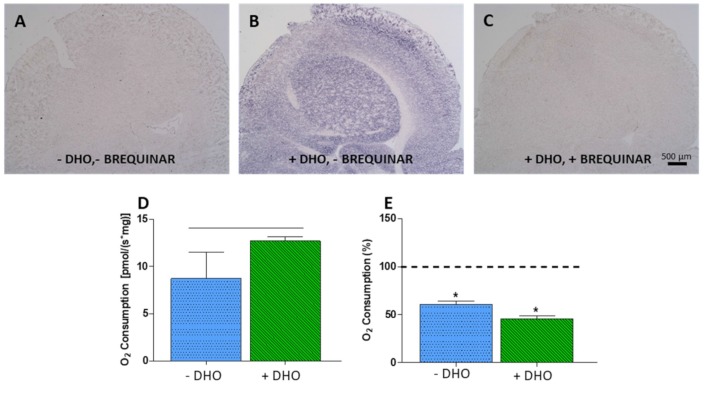
**DHODH activity.** (**A**–**C**) Histochemical detection of DHODH activity in adult mouse brain. (**A**) Negative control, no dihydroorotate (DHO) added. (**B**) DHODH activity, DHO added. (**C**) DHODH activity inhibition, DHO and its inhibitor Brequinar (20 μM) added. (**D**) Oxygen consumption increment in adult mouse brain after addition of DHO; horizontal black line indicates significant difference. (**E**) Inhibition of oxygen consumption by leflunomide in brain homogenate with (green) and without (blue) addition of DHO. Dashed line (100 %) indicates mean values of oxygen consumption in uninhibited cells. Bars indicate oxygen consumption in leflunomide inhibited cells N = 3. *: p < 0.05 (versus uninhibited cells).

DHODH histochemistry analysis in adult rat brain showed high staining in different regions, with the hippocampus presenting a characteristic pattern [38]. We could not get optimally-cryopreserved adult human brain samples appropriate for histochemical studies. However, we detected high DHODH activity in adult mouse brain homogenate and Brequinar, a DHODH inhibitor, largely reduced the staining (Figure 7A–7C). Moreover, fresh samples from adult mouse brain consume significantly more oxygen when dihydroorotate was used as electron donor (Figure 7D). Leflunomide reduces oxygen consumption (Figure 7E). Similar to proliferating SH-SY5Y cells, in the absence of dihydroorotate, leflunomide was able to reduce oxygen consumption to a residual 60 %. This result highlights the importance of *de novo* pyrimidine biosynthesis pathway in adult brain, not only for pyrimidine production, but also for OXPHOS function.

Supporting our results on adult brain DHODH activity, in the 1960s and 1970s, different publications showed the presence of the *de novo* pyrimidine biosynthesis pathway in adult mouse, rat, and cat brain. Thus, the intracranial injection of radioactive intermediates for this pathway resulted in an early appearance of radioactive pyrimidine nucleotides in the brain [39–41]. Moreover, administration of these radioactive compounds to adult rat brain sections or homogenates also produced radioactive pyrimidine nucleotides [40, 42–48]. Finally, high DHODH activity was found in adult rat brain homogenate [49].

### DHODH immunohistochemistry

Previous results strongly suggested the presence of the *de novo* pyrimidine biosynthesis pathway in adult human brain. However, these results did not indicate in which cell type this route was expressed. Immunocytochemical staining with anti-DHODH antibody confirmed DHODH in frontal cortex, hippocampus, striatum, and substantia nigra pars reticulate in the adult rat brain. Immunoreactivity was found in neuronal bodies and absent in neuroglia [38]. Moreover, neuronal incorporation of radioactive orotic acid intraventricularly injected into adult rat brain was higher than into glial cells [50].

**Figure 8:**
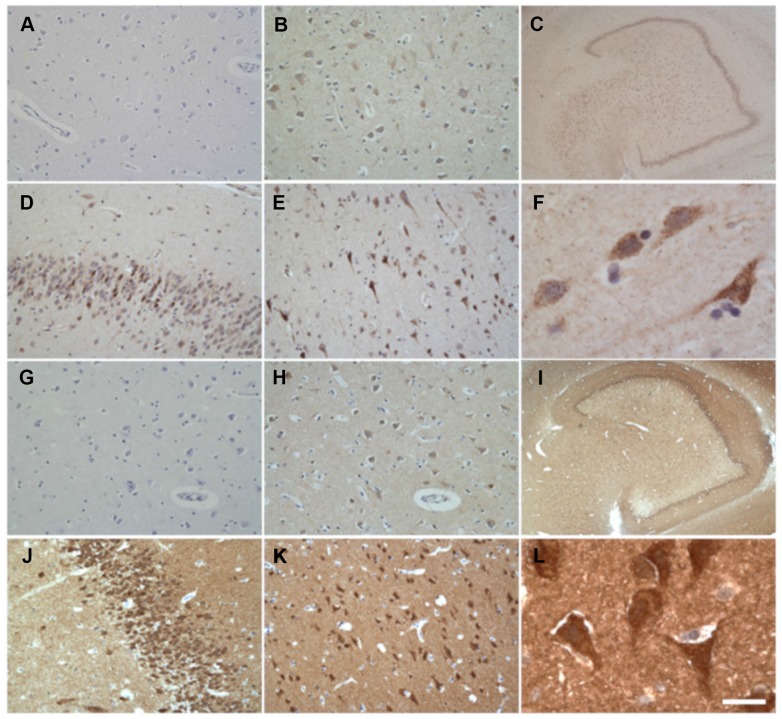
Immunohistochemical detection of neuronal CAD (**A**–**F**) and DHODH (**G**–**L**) in adult non-AD human brain. (**A**, **G**) Negative control. No primary antibody. (**B**, **H**) Positive control. (**C**, **I**) Dentate gyrus. (**D**, **J**) Granular layer of dentate gyrus. (**E**, **K**) Frontal cortex. (**F**, **L**) Entorhinal cortex. Scale bar in **L** represents 1000 μm in **C** and **I**; 100 μm in **A**, **B**, **D**, **E**, **G**, **H**, **J** and **K**; and 20 μm in **F** and **L**.

Immunohistochemistry staining of adult human samples with anti-CAD and anti-DHODH antibodies confirmed the presence of both enzymes in different brain regions, such as amygdala, cerebellum, DG, EC, frontal cortex, putamen, and striatum (Figure 8). Stronger staining was observed in zones with high neuronal density whereas the immunoreactivity was hardly seen in white matter. Furthermore, no marking is noted in cytoplasm of glial cells (Figure 8). In neurons, staining is located in perikarya and dendrites (Figure 8 and Supplementary Figure 5). Supporting our results, the Human Protein Atlas Webpage (https://www.proteinatlas.org/, accessed April 26, 2018) shows that CAD and DHODH proteins appear, at low levels, in different adult brain regions, such as cerebral cortex, hippocampus, caudate, and cerebellum. Neurons and glial cells showed medium-low and low or no staining, respectively.

Although CAD is a cytosolic enzyme and DHODH is a mitochondrial one, the pattern of staining is very similar for both of them. These enzymes together with UMPS, another cytosolic enzyme, belong to the same metabolic pathway, and it has been shown that CAD and UMPS are placed around and outside mitochondria, closely associated with their outer membranes [5, 51]. Then, the enzymes for the complete biosynthetic pathway are physically associated in the cell.

All these results confirm the presence of the *de novo* pyrimidine biosynthesis in mature neurons from adult human brain. We wondered if this biochemical pathway would be affected in Alzheimer disease patients.

### Pathways for pyrimidine nucleotide synthesis are altered in brains from Alzheimer disease patients

### UCK2 and DHODH mRNA levels in different Alzheimer disease stages

To study the biochemical pathways for pyrimidine nucleotide synthesis in AD patients, we analyzed *UCK2* and *DHODH* mRNA levels from different brain regions, such as LC, EC, hippocampal cornus ammon 1 (CA1), and DG. Neurofibrillary pathology is found in LC and EC in AD Braak and Braak stages I/II, CA1 in AD stages III/IV, but not in DG, even in AD stages V/VI [52].

**Figure 9:**
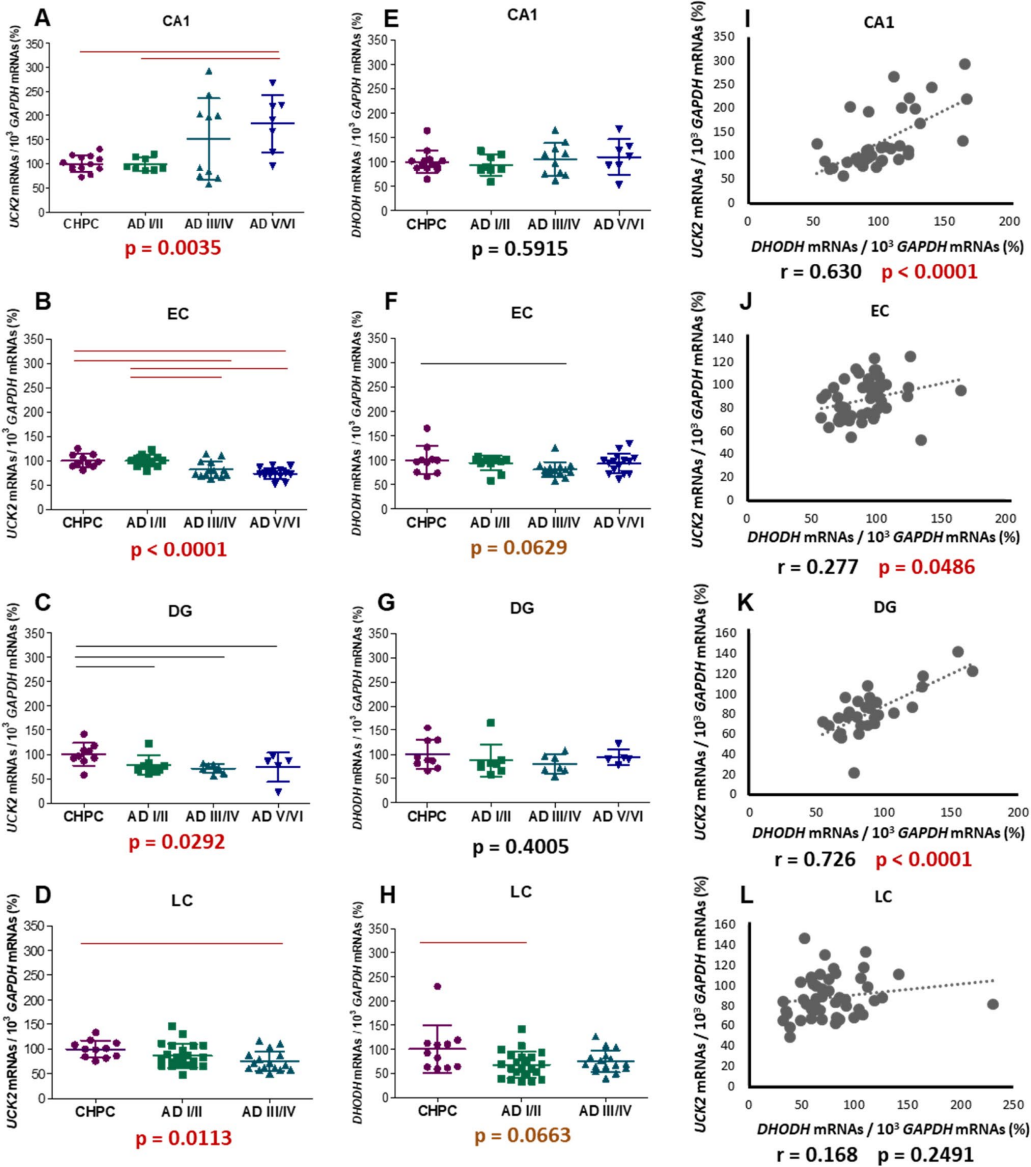
**mRNA levels of selected genes encoding proteins from both pyrimidine nucleotide synthesis pathways.** Graphs represent mean ± standard deviation values of *UCK2* (**A**–**D**), *DHODH* (**E**–**H**), and *DHODH* - *UCK2* correlations (**I**–**L**); respectively. *GAPDH* mRNA levels have been used to normalize. CA1: hippocampal cornus ammon 1; EC: entorhinal cortex; DG: dentate gyrus; LC: locus ceruleus. Each point represents an individual sample. The p values for Kruskal-Wallis or ANOVA tests are indicated under the graphs. Red and black horizontal lines indicate significant p values, fulfilling (p < 0.0083, p < 0.0167 in LC) or no (p ≤ 0.05) the post-hoc Bonferroni/Dunn criteria.

AD stages were significantly associated with *UCK2* mRNA levels in all these brain areas (Figure 9A–9D). However, the pattern was different in CA1, where higher levels were found in more severe AD stages, than in the other regions, where *UCK2* mRNAs tended to decrease as neuropathology progressed. A potential explanation, already pointed out to justify lower CA1 mitochondrial DNA (mtDNA) deletion levels [53], would be that a selective and progressive apoptosis of CA1 neurons with decreasing *UCK2* mRNA levels may shift the pattern towards an apparent increase of ‘healthy’ cells. In contrast, *DHODH* mRNA levels were not associated with AD stages (Figure 9E–9H). These results resemble those previously found in KCN-treated SH-SY5Y cells, in which energy stress provoked a decrease in *UCK2* mRNA levels but did not modify *DHODH* mRNA levels (Figure 4D), and the same explanation might be advanced. Despite this, and except for LC, positive correlations between *DHODH* and *UCK2* mRNA levels were observed in these regions (Figure 9I–9L), thus suggesting that both pathways are essential.

Associations between these mRNA levels and AD stages are not due to unbalanced sex or postmortem delay distributions. No association was found between AD stages and RNA integrity number (RIN) values. AD stages and age are significantly associated [54]. Although there were no significant correlations between age and *DHODH* mRNA levels, age and *UCK2* mRNA levels were significantly correlated (Supplementary Figure 6A–6H). These correlations were negative in LC, EC, and DG, and positive for CA1. To evaluate the relevance of AD stages versus age on *UCK2* mRNA levels, we divided individuals according to their *UCK2* mRNA levels (higher and lower half) for each brain region and AD stage, and compared their ages (Supplementary Figure 6I–6L). Except for DG AD I/II stages, there were no significant differences. This result suggests that variation in *UCK2* mRNA levels is more dependent on AD stage than on age.

If lower *UCK2* mRNA levels in different brain regions correspond with a decrease in pyrimidine synthesis pathway, and, as it has been recently reported, AD subjects show lower uridine concentrations in plasma and cerebrospinal fluid [55–59], then AD patients probably have a cerebral deficiency in many cellular functions related to the metabolism of uridine. Therefore, it can be suggested that uridine therapeutic administration would improve biochemical pathways related to brain function. In this line, uridine treatment attenuates the impairments in learning and memory observed in gerbil, stressed rat, and AD mouse models [27, 60–63]. Nutrient combinations including uridine supplementation have shown promising results in AD therapy [64–69].

As previously proposed, reduction in *UCK2* mRNA levels might be secondary to a drop in energy production. OXPHOS is the main energy provider to power neuronal activity [70]. Thus, OXPHOS dysfunction in AD patients might explain the brain reduction in *UCK2* mRNA levels.

### Reduction in MT-CO1 and COX4I1 mRNA levels is associated with an increase in DHODH/UCK2 ratio in entorhinal cortex from Alzheimer disease patients

**Figure 10:**
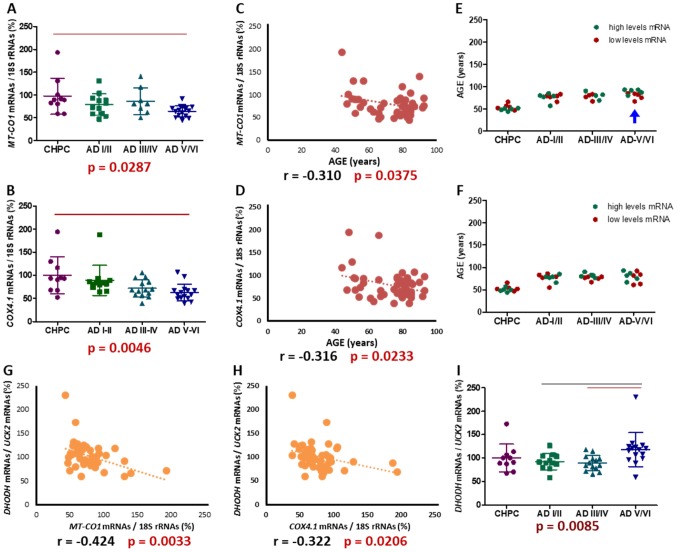
**Entorhinal cortex *MT-CO1* and *COX4I1* mRNA levels.** (**A**, **B**) *MT-CO1* and *COX4I1* mRNA levels, normalized by 18S rRNA, in different AD stages. Points represent individual samples and horizontal lines indicate mean ± standard deviation values. The p value for Kruskal-Wallis test is indicated under the graph. Red line indicates p < 0.0083 (fulfilling the post-hoc Bonferroni/Dunn criteria). (**C**, **D**) Correlations between age and *MT-CO1* or *COX4I1* mRNA levels. (**E**, **F**) Age distribution, according to *MT-CO1* or *COX4I1* mRNA levels, in different AD stages. Green and red dots indicate higher and lower half *MT-CO1* or *COX4I1* mRNA levels, respectively. Blue arrow indicates significant differences, in age, between individuals with higher and lower *MT-CO1* mRNA levels. (**G**, **H**) Correlations between *MT-CO1* or *COX4I1* mRNA levels and *DHODH*/*UCK2* ratio. (**I**) *DHODH*/*UCK2* ratio in different AD stages. Points represent individual samples and horizontal lines indicate mean ± standard deviation values. The p value for Kruskal-Wallis test is indicated under the graph. Black and red lines indicate p ≤ 0.05 or, fulfilling the Bonferroni/Dunn criteria, p < 0.0083.

We used mtDNA-encoded *MT-CO1* and nuclear DNA (nDNA)-encoded *COX4I1* mRNA levels as surrogates of OXPHOS function. A comparison of mRNA levels and AD stages in different brain regions showed a significant association in EC (Figure 10A, 10B). In this region, AD stages V/VI patients had lower *MT-CO1* and *COX4I1* mRNA values than controls. The *COX4I1* mRNA levels were also associated to AD stages in DG and LC. We noted above that AD stages and age are significantly associated. Moreover, it was previously reported that *MT-CO1* mRNA levels were significantly lower in cerebral hemispheres from 28 than 9 months old rats [71]; and the mRNA levels for the three mtDNA-encoded CIV subunits were lower in cerebral cortex from 24- than 18-month-old mice [72]. We have also observed a negative significant correlation between *MT-CO1* and *COX4I1* mRNA levels and age, but only in EC (Figure 10C, 10D). To evaluate the importance of AD stages versus age on EC *MT-CO1* and *COX4I1* mRNA levels, we divided individuals according to their *MT-CO1* and *COX4I1* mRNA levels (higher and lower half) for each AD stage, and compared their ages (Figure 10E, 10F). Except for EC AD V/VI stages, there were no significant differences. However, contrary to what would be expected, and what is observed in the correlation, the youngest individuals had lower *MT-CO1* mRNA levels. This result suggests that variation in *MT-CO1* and *COX4I1* mRNA levels is more dependent on AD stage than on age.

A decrease in mRNA levels for mtDNA-encoded CIV subunits has also previously been described in some brain regions of AD patients [73–82]. Furthermore, low mRNA levels from nDNA-encoded CIV genes were informed in AD patients [82–86]. This reduction in mRNA levels for CIV subunits is probably responsible for the widely reported brain CIV deficit in many AD patients [75, 87–108].

Interestingly, in EC, *MT-CO1* and *COX4I1* mRNA levels negatively correlate with *DHODH*/*UCK2* ratio (Figure 10G, 10H). In EC, this ratio was associated with AD stages (Figure 10I), and was significantly higher in AD stages V/VI than that in I/II and III/IV. The ratio did not correlate with age. The higher *DHODH*/*UCK2* ratio in EC of AD stages V/VI is perhaps an attempt to compensate for a reduction in energy production by a CIV defect. As down-regulation of the *de novo* pathway might affect many vital reactions, because of its importance for OXPHOS function, a reduction in the salvage pathway, to avoid dispensable ATP consumption, would cause a higher *DHODH*/*UCK2* ratio.

As a conclusion, our results confirm that an OXPHOS dysfunction, throughout an altered *de novo* pyrimidine biosynthesis pathway, can affect variables of post-mitotic cells, such as neuronal differentiation; that the *de novo* pyrimidine biosynthesis pathway is present in adult human brain; and that both *de novo* and salvage pyrimidine nucleotide synthesis pathways appear to be unbalanced in AD brain. However, more work is required to link these pathways for pyrimidine nucleotide synthesis to an OXPHOS defect in AD brain. In particular, the determination of pyrimidine nucleotide levels and DHODH and UCK2 activities in large numbers of AD brains could confirm our RNA results.

## METHODS

### Cells, culture conditions, and differentiation

The human neuroblastoma SH-SY5Y cell line was obtained from Sigma-Aldrich (catalogue number 94030304, lot 13C014, P 17; St. Louis, MO, USA). These cells were cultured in Dulbecco’s modified Eagle’s medium (DMEM) containing 25 mM glucose, 4 mM L-glutamine, and 1 mM sodium pyruvate, supplemented with 10 % fetal bovine serum (FBS). Differentiation was induced following optimized protocols [109], but instead of 25 mM glucose, we used 5 mM galactose media to enhance OXPHOS function. Cells were maintained at 37 °C in a humidified atmosphere of 5 % CO_2_.

Three growth curves were performed for each condition, and each time point (from day 0 to day 12) was counted in triplicate.

### Human and mouse brains

Brain samples from three healthy adult individuals were obtained from the Institute of Neuropathology HUB-ICO-IDIBELL Biobank following the guidelines of Spanish legislation on this matter and the approval of the local ethics committee.

Mouse brain was obtained from C57BL/6J adult individuals (2 to 8 months) after the approval of the local ethics committee. Animals were killed by decapitation and the brain was immediately removed. For histochemistry analysis, brains were covered with optimal cutting temperature (OCT) compound and frozen at - 80 °C. For oxygen consumption studies, brains were used immediately after dissection.

### RNA analysis

Brain RNA samples were obtained from the Institute of Neuropathology HUB-ICO-IDIBELL Biobank. The post-mortem interval between death and tissue processing was between 2 and 22 h 15 min. Processing of brain tissue has been detailed elsewhere [110]. Neuropathological diagnosis and staging of AD were based on the classification of Braak [52]. CHPC were middle-aged individuals (n = 20, 11 men, 9 women; age, 50.7 ± 7.2 years) with no neurological or mental disorders and without lesions on the neuropathological study. Four brain regions were examined: CA1, DG, EC, and LC. A summary of all analyzed individuals is provided (Supplementary Table 2). Not all regions were assessed in every case because of the limited availability of tissues.

### RNA purification

RNA from frozen cells and tissue was extracted following the instructions of the supplier (NucleoSpin RNAII, Macherey-Nagelsupplier, Düren, Germany and RNeasy Mini Kit, Qiagen, Hilden, Germany). RIN and 28S/18S ratios were determined with the Agilent Bioanalyzer (Agilent Technologies, Inc., Santa Clara, CA). Samples were digested with DNase, and RNA concentration was evaluated using NanoDrop Spectrophotometer (Thermo Fisher Scientific, Waltham, MA).

### qPCR

The mRNA levels were measured with the RT-qPCR method using an Applied Biosystems StepOne^TM^ Real-Time PCR System Thermal Cycling Block and a ViiA 7 Real-Time PCR system. *DHODH*, *UCK2* and *MT-CO1* mRNA levels were determined in triplicate with RT-qPCR using TaqPath^TM^ 1-Step Multiplex Master Mix (*DHODH* and *UCK2*) and TaqMan^®^ RNA-to-C_T_^TM^ 1-Step Kit (*MT-CO1*). The expression levels were normalized using *GAPDH* mRNA or *18S* rRNA. Serial dilutions of recombinant plasmid DNA were included in each experiment to generate a standard curve for *DHODH,* *UCK2*, and *GAPDH*.

### Western blot and ELISA

For immunoblot analysis, protein levels were analyzed in whole-cell lysates obtained using cell lysis buffer (RIPA) or in tissue homogenates obtained using glass potters at 500 rpm, with 10 strokes, and Tris-buffered saline (TBS). Samples were resolved on SDS-PAGE minigels (Miniprotean, Bio-Rad, Hercules, CA, USA) and were transferred to PVDF membranes (Trans-Blot^®^ Turbo™ Mini PVDF Transfer Pack, Bio-Rad) using a Trans-Blot^®^ Turbo^TM^ Blotting System (Bio-Rad). Membranes were analyzed by immunoblotting with the following antibodies: rabbit polyclonal anti-DHODH (1:500) from biorbyt (orb247660, Cambridge, UK), rabbit mono and polyclonal anti-CAD (1:500), mouse monoclonal anti-DHODH (1:1,000), mouse polyclonal anti-UCK2 (1:250) from Abcam (ab40800, ab99312, ab54621, ab167683, Cambridge, UK), mouse anti-actin (1:1,000) from Sigma (A5441, St. Louis, MO, USA), and rabbit anti-CII (1:1,000) from Thermo Fisher Scientific (459200, Waltham, MA, USA). After washing, the membrane was incubated with peroxidase-conjugated secondary antibodies (1:5,000 or 1:10,000) from Thermo Fisher Scientific for 1 h at room temperature or it was incubated with appropriate secondary antibodies DyLight™ (SA535521, SA535571, 35568, 35518, Thermo Fisher Scientific). Bands were visualized with Super Signal West Pico Chemiluminescence Substrate from PIERCE^®^ (Thermo Fisher Scientific) or using Odyssey^®^ CLx Imaging System (LI-COR Biosciences, San Jose, CA, USA).

The DHODH peptide corresponding to amino acids 32–141 was heterologously expressed in *Escherichia coli strain* C41 (DE3) as a recombinant peptide using pET28a expression vector.

The quantitative determination of DHODH and UCK2 proteins was performed using commercially available Human Dihydroorotate Dehydrogenase (DHODH) ELISA kit and Human Uridine-Cytidine Kinase 2 (UCK2) ELISA kit (Abbexa, Cambridge, UK). Protein extracts concentrations were measured by Bradford assay. Samples were collected and DHODH and UCK2 levels were immediately measured, following the manufacture’s instructions.

### Histochemistry and immunohistochemistry

### Immunocytochemistry

Cells were fixed with 4 % paraformaldehyde for 15 min at room temperature and permeabilized using 0.1 % Triton X-100 (Sigma-Aldrich) diluted in PBS for 10 min. To block unspecific epitopes, cells were incubated with 0.1 % bovine serum albumin. Primary antibodies (rabbit anti-TUBB3, 1:1,000, from Abcam and rabbit anti-TH, 1:200, from Sigma-Aldrich) were incubated overnight at 4 °C followed by incubation with appropriate fluorescently labeled secondary antibodies, Alexa Fluor® 488 and 594 (Molecular Probes, Eugene, Oregon, USA) for 1 h at room temperature. Finally, cell nuclei were counterstained with 4',6-diamidino-2-phenylindole (DAPI) (Sigma-Aldrich).

### Histochemistry

DHODH histochemistry was performed using the nitroblue tetrazolium/formazan technique on 20 μm cryostat sections of adult mouse brain. The oxidation of its substrate dihydroorotate (DHO) (10 mM) can be determined by trapping the electrons with tetrazolium (1 mM) in the presence of cyanide (10 mM), to prevent their further flow along the chain to oxygen, in phosphate buffer pH 8, 37 °C, 60 min. The insoluble blue product formazan can be revealed by light microscopy [38].

### Immunohistochemistry

This protocol was performed at the Anatomic Pathology Core Unit of the Health Sciences Institute of Aragon (Zaragoza, Spain). Once paraffin blocks were made, 2.5 μm sections were cut with a rotation microtome (Leica RM2255) and paraffin sections were taken on superfrost glass slides. Slides were air dried at 37 °C overnight and, for immunohistochemistry stain, they were deparaffinized in xylene for 10 min, rehydrated in a grades series of ethanol (100, 100, 96, 70, 5 min each) and distilled water for 5 min. After rehydration, antigen retrieval was performed by means of the PT Link (Dako) at 95 °C for 20 min in a low/high pH buffer (Dako antigen retrieval, low/high pH). After retrieval, automated immunostaining was performed with a previously optimized protocol. For this process endogenous peroxidase was first blocked (EnVision FLEX Peroxidase-Blocking) followed by 5 min 2 washing steps (Dako wash buffer); mouse monoclonal anti-DHODH 1/50 and rabbit polyclonal anti-CAD 1/200 (ab54621, ab99312, Abcam, Cambridge, UK) primary antibodies were used with 40 min incubation time. After 2 wash steps a dual rabbit/mouse HRP conjugated visualization system for signal amplification was used (Envision Flex HRP, Dako). After 3 wash steps (Dako wash buffer, 5 min each), 3,3'-Diaminobenzidine (DAB) was used for signal development after precipitation at primary antibody binding sites.

The double staining of CAD, DHODH and UCK2 with NeuN and GFAP, was performed using commercially available PolyStain TS kit (NB-23-00131, Neo-Biotech, Nanterre, France). Anti-CAD, anti-DHODH and anti-UCK2 antibodies (ab99312, ab232767, ab60222, from Abcam) were incubated overnight at 4 °C at 1/50. Anti-NeuN (ab104224, Abcam) and anti-GFAP (G3893, Sigma) antibodies were incubated 2 h at 1/100.

### Flow cytometry

Cell suspensions were first fixed with 4 % paraformaldehyde for 15 min at 4 °C and permeabilized using a commercial buffer (Thermo Fisher Scientific). Samples were incubated overnight with the primary antibodies rabbit anti-TUBB3 (1:1,000) from Abcam and rabbit anti-TH (1:200) from Sigma, and then washed with PBS (Thermo Fisher Scientific). Cells were incubated for 1 h with appropriate secondary antibodies Alexa 488 (Thermo Fisher Scientific), washed, and then analyzed on a BD FACScan System (Becton-Dickinson, San Jose, CA, USA). 10,000 cells were studied. The results were analyzed using Weasel software.

### Oxygen consumption

Neuroblastoma SH-SY5Y cell line: Cells were cultured in 5 mM galactose media. The cells were then collected by trypsinization, washed, counted, and resuspended at 1.5 x 10^6^ cells/ml. Endogenous and inhibited respiration (with leflunomide or cyanide) analyses were performed. Each condition was analyzed three times.

### Mouse brain

Brain was immediately obtained after animal death, dissected on ice, and weighed on an analytical balance. The dissected brain was directly transferred into ice-cold mitochondrial respiration medium (MIR05: 110 mM sucrose, 60 mM K^+^-lactobionate, 0,5 mM EGTA, 3 mM MgCl_2_, 20 mM taurine, 10 mM KH_2_PO_4_, 20 mM HEPES and 1 g/l BSA, pH 7.1). Tissues were homogenized in the same medium with a pre-cooled glass potter at 1,000 rpm, 16 strokes. Resulting homogenates containing 10 mg tissue wet weight were suspended in 1 ml of ice-cold MIR05. To measure the oxygen consumption in the presence of the substrate, MIR05 medium contained 6 mM of DHO. Endogenous and inhibited respiration (with leflunomide) analyses were performed. Each condition was analyzed three times.

Oxygen consumption of cells and mouse brain was analyzed using the high-resolution oxygraph OROBOROS^®^.

### Mass spectrometry analysis of protein spots

### Protein identification by peptide mass fingerprint

 Spots were excised from gels, reduced, alkylated and digested with trypsin sequencing grade (Roche Molecular Biochemicals) [111]. Produced peptides were analyzed in a 4800 Plus Proteomics Analyzer MALDI-TOF/TOF mass spectrometer (Applied Biosystems, MDS Sciex, Toronto, Canada) at the Proteomics Unit of Complutense University of Madrid. The MS data was searched against SwissProt Data Base with taxonomy restriction to human (553,231 sequences) using MASCOT 2.3 (http://www.matrixscience.com) search engine through Global Protein Server v 3.6 software (ABSciex). The search parameters were carbamidomethyl cysteine as fixed modification and oxidized methionine as variable modification. Peptide mass tolerance was 50 ppm and up to 1 missed trypsin cleavage site allowed. All identified protein outperformed the probability scores fixed by mascot as significant with a p-value minor than 0.05.

### Protein identification by PRM

Desalted peptides from digested protein were analyzed by targeted proteomics to identify only the protein of interest in this study (PYRD or DHODH). The software Skyline 4.1 was used to build and optimize the PRM method for detection of unique peptides from DHODH [112]. An inclusion list consisted of m/z of resultant target candidates for each peptide was exported to Xcalibur 4.0 (Thermo Scientific) acquisition software of Q-Exactive HF mass spectrometer. The final PRM methods included one protein, 49 peptides and 112 precursors. The peptides for PRM were analyzed by nanoflow liquid chromatography-tandem mass spectrometry in an EASY-nLC 1000 System coupled to the Q-Exactive HF mass spectrometer through the Nano-Easy spray source (all from Thermo Scientific, Bremen, Germany). MS Data acquisition was performed in Q-Exactive HF using PRM method. Then Data were analyzed in Skyline software and identified with Mascot search engine thorough Proteome discoverer 2.2 software (Thermo Scientific). Peptides identifications based on MSMS data were used by skyline to generate a spectral library. To confirm DHODH detection, data were processed against the PRM-library on Skyline and manually inspected to ensure consistency between the transitions detected and the sequences of peptide searched.

### Statistical analysis

The statistical package StatView 6.0 and GraphPad Prism 5.0 were used to perform all the statistical analyses. The normality of distribution was analyzed with Kolmogorov-Smirnov test. Mann-Whitney or Kruskal-Wallis non-parametric tests were used when values did not follow a normal distribution, while the ANOVA test was used for normal variables. To compare more than two groups post-hoc tests were also performed. All data were expressed as mean ± standard deviation and significance levels were set at p < 0.05 and the levels indicated by the post-hoc tests. Statistical values in cell lines were obtained after three or more independent experiments. In each independent experiment, several technical replicates were performed.

## Supplementary Material

Supplementary Figures

Supplementary Tables 1 and 3

Supplementary Table 2
