# Supplementary material for: Brain pyrimidine nucleotide synthesis and Alzheimer disease
Source: Aging (Albany NY). 2019 Sep 27;11(19):8433–62. doi: 10.18632/aging.102328 (PMC6814620; doi:10.18632/aging.102328)
Supplement: Supplementary Figures [file aging-11-102328-s003.pdf]

SUPPLEMENTARY FIGURES

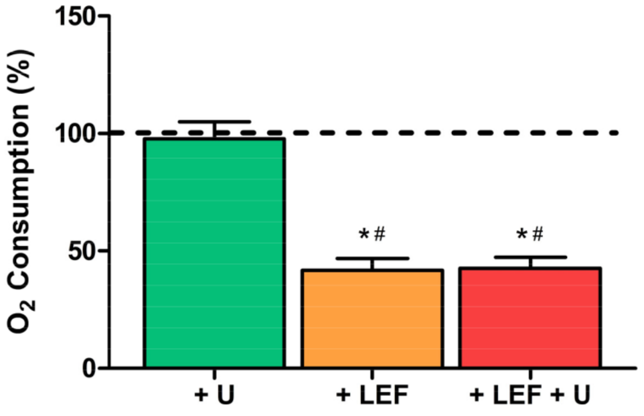

Supplementary Figure 1. Effect of uridine (U) on oxygen consumption of leflunomide (LEF)-untreated and treated SH-SY5Y cells. Dashed line (100 %) represents mean values of untreated cells. Bars indicate main values and standard deviations of U, LEF and LEF+U treated cells. *: $p < 0.05$ (versus untreated cells); #: $p < 0.05$ (versus U-treated cells).

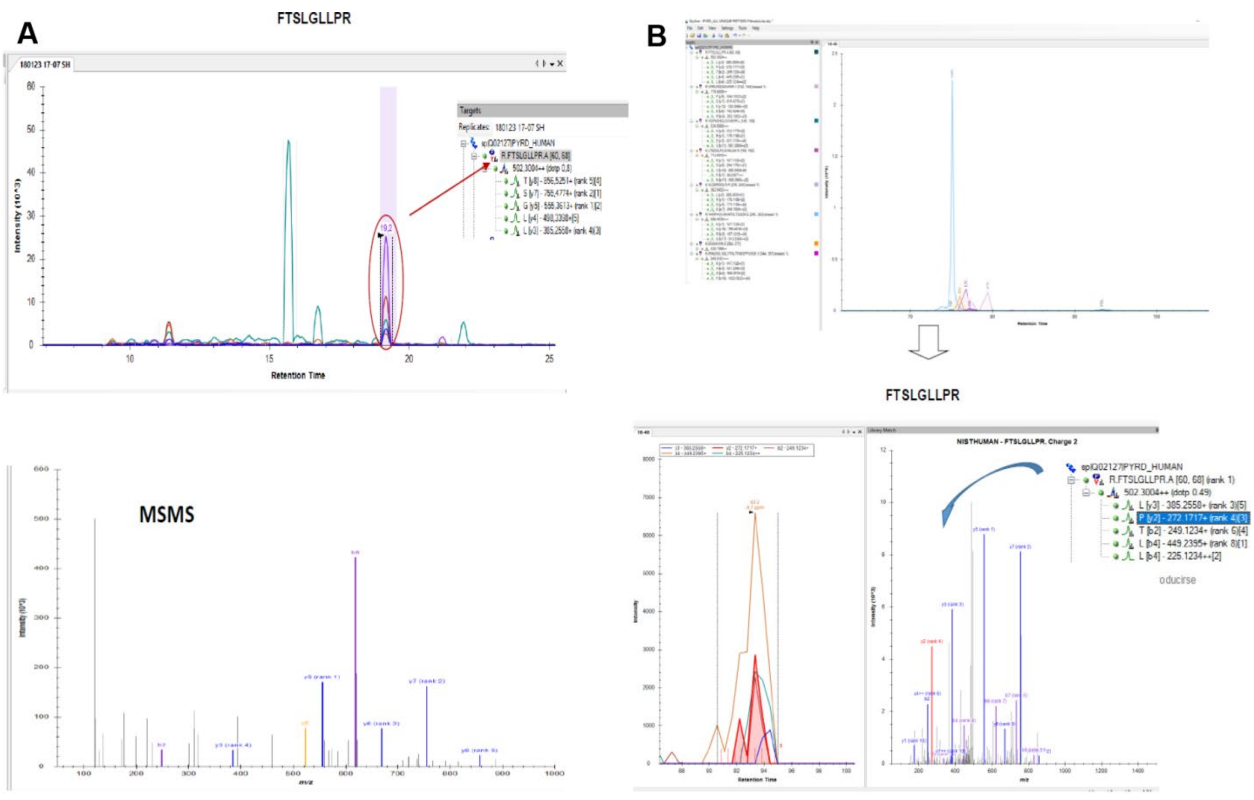

Supplementary Figure 2. Results of the parallel reaction monitoring proteomic analysis. MS/MS spectrum of the FTSLGLLPR peptide. (**A**) Neuroblastoma SH-SY5YH cell line homogenate from the candidate western blot band. (**B**) Adult human brain homogenate from the candidate 2D-western blot spot.

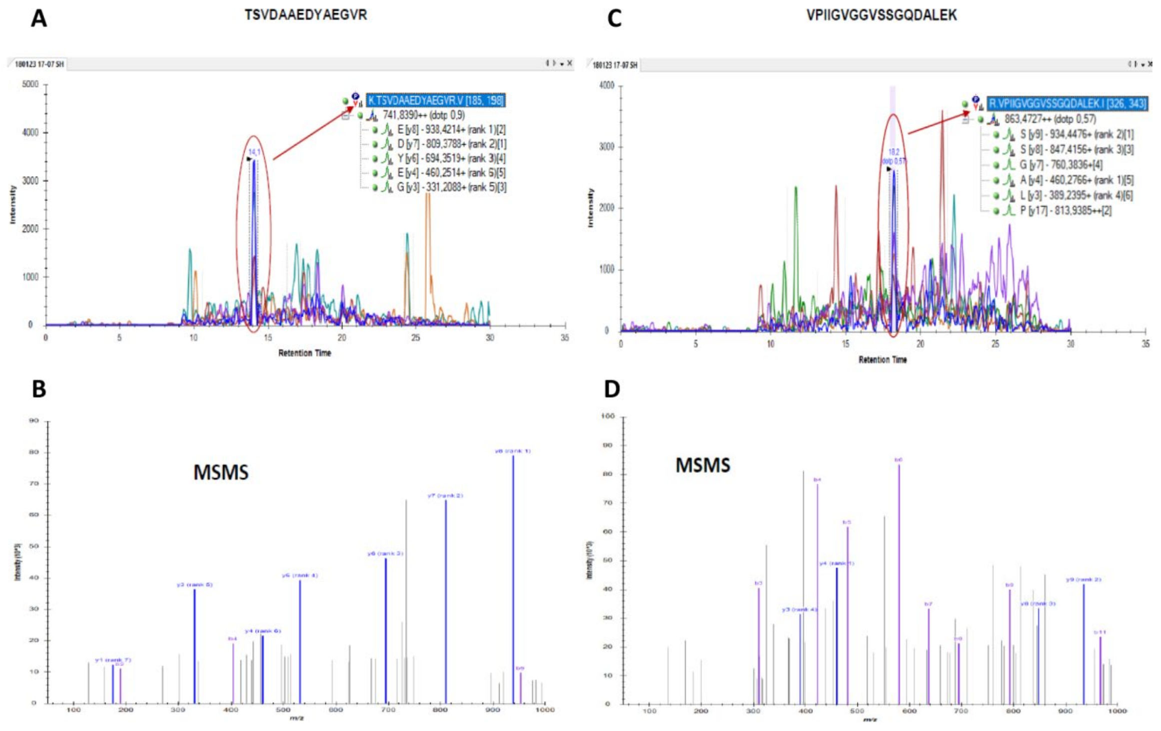

Supplementary Figure 3. Results of the parallel reaction monitoring proteomic analysis for the candidate western blot band from the neuroblastoma SH-SY5YH cell line homogenate. MS/MS spectra of the TSVDAAEDYAEGVR (**A**, **B**) and VPIIGVGGVSSGQDALEK (**C**, **D**) peptides.

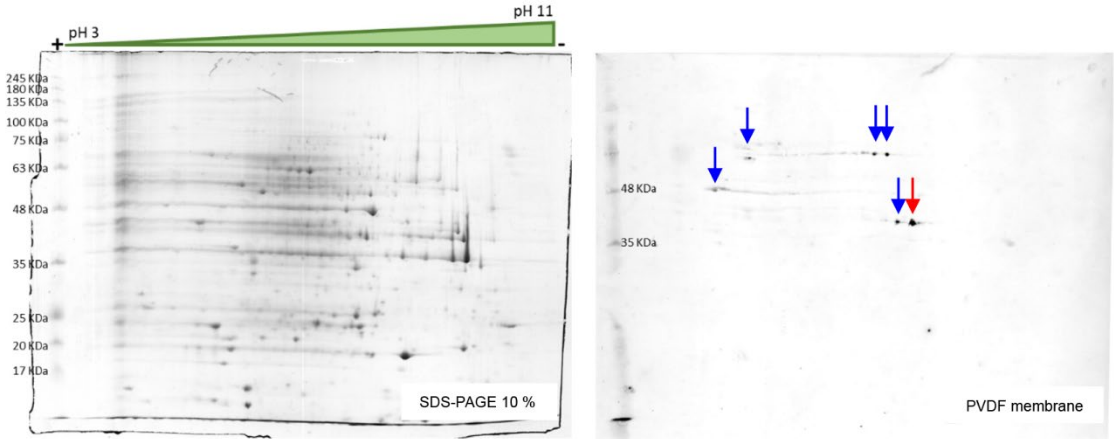

Supplemental Figure 4. 2D-western blot analysis of the adult human brain. (**A**) SDS-PAGE 10 % results. (**B**) PVDF membrane showing the selected spots for peptide mass fingerprinting (blue and red arrows) and parallel reaction monitoring (PRM) (red arrow) proteomics analyses.

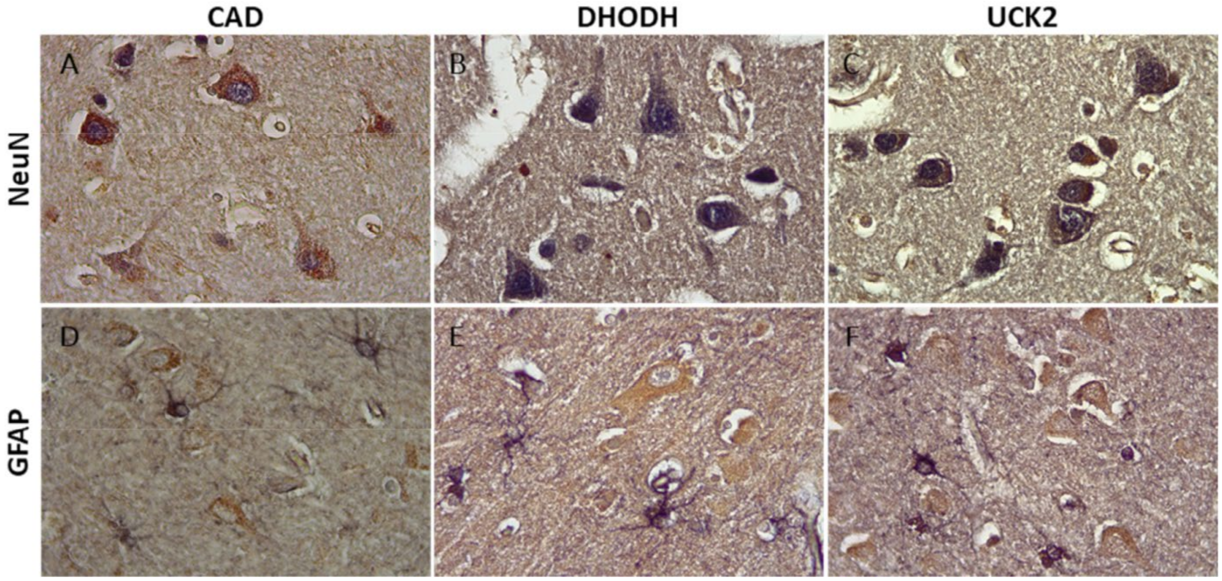

Supplementary Figure 5. Neuronal location of enzymes for *de novo* pyrimidine biosynthesis (CAD and DHODH) and salvage (UCK2) pathways. Representative immunohistochemical images of CAD (**A**, **D**), DHODH (**B**, **E**) and UCK2 (**C**, **F**) in frontal cortex of human brain, where neuronal cytoplasms are stained brown. The neuronal nucleus (NeuN, blue stain, **A**–**C**) and astrocyte cytosol (GFAP, blue stain, **D**–**F**) markers are also indicated. (63 x).

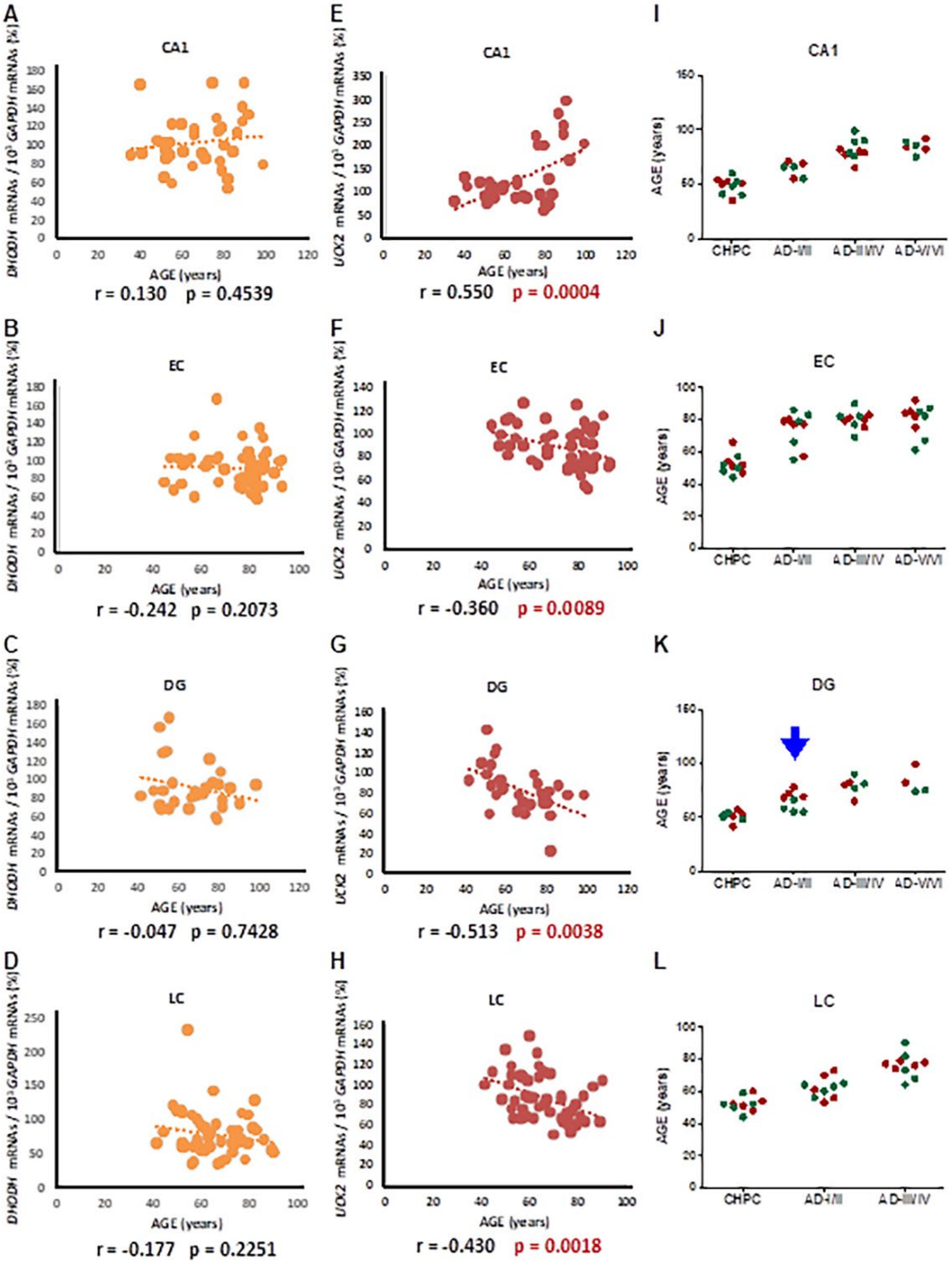

Supplementary Figure 6. mRNA expression by age and AD stages. Correlation between *DHODH* (**A**–**D**) or *UCK2* (**E**–**H**) mRNA levels with age. *GAPDH* mRNA levels have been used to normalize. Correlation coefficients and p values are shown in the graphs. (**I**–**L**) Age distribution, according to *UCK2* mRNA levels, in different AD stages. Green and red dots indicate higher and lower half *UCK2* mRNA levels, respectively. Blue arrow in panel **K** indicates separation by age between individuals with higher and lower *UCK2* mRNA levels. CA1: hippocampal cornus ammon 1; EC: entorhinal cortex; DG: dentate gyrus; LC: locus ceruleus.
