# Supplementary material for: Brain pyrimidine nucleotide synthesis and Alzheimer disease
Source: Aging (Albany NY). 2019 Sep 27;11(19):8433–62. doi: 10.18632/aging.102328 (PMC6814620; doi:10.18632/aging.102328)
Supplement: Supplementary Tables 1 and 3 [file aging-11-102328-s002.pdf]

SUPPLEMENTARY TABLES

Supplementary Table 1. Brain expression of genes for the *de novo* pyrimidine biosynthesis pathway, and *UCK2*, from different animal species.

Animals	*CAD*	*DHODH*	*UMPS*	*UCK2*
Chlorocebus sabaeus[a,b,c]	4-32	1-10	6-24	5-18
Macaca mulatta[a,b,c]	14-15	14-18	6	16-29
Papio anubis[a,b,c]	8-13	5-9	9-14	13-32
Mus musculus[a,b]	6-27	6-10	11-30	13-71
Rattus norvegicus[a,b]	4-11	4-6	22-34	11-26
Bos taurus[a,b]	8-9	-	6	42
Equus caballus[a,b]	6	7	53	26
Ovis aries[a,b]	1	4	4	7
Monodelphis domestica[a,d]	23	32	39	22
Gallus gallus[e]	8-9	6-10	8-12	62-79
Xenopus tropicalis[f]	33	25	18	12

Expression is reported as number of transcripts of a particular gene per million molecules in the RNA-seq sample. a, mammal; b, placental; c, primate; d, marsupial; e, bird; f, amphibian.

Please browse Full Text version to see the data of Supplementary Table 2.

Supplementary Table 2. Summary of cases used for mRNA expression studies. CA1: hippocampal cornus ammon 1; DG: dentate gyrus; EC: entorhinal cortex; LC: locus ceruleus. CHPC: Clinical and HistoPathological Controls. RIN: RNA integrity number. F: female; M: male. PMD: post-mortem delay. *DHODH* and *UCK2* mRNA levels have been normalized to 10^3 *GAPDH* mRNAs. The CHPC *MT-CO1* / *18S* ratio mean values have been considered 100 %.

Supplementary Table 3. Expression of genes for the *de novo* pyrimidine biosynthesis pathway, *UCK2* and *GAPDH*, in different regions from human adult brain.

Brain region	*CAD*	*DHODH*	*UMPS*	*UCK2*	*GAPDH*	Sex-Age	STUDY
Amygdala	1	6	11			F60, F76	68 FANTOM
Brain	1	4	7			M18	68 FANTOM
Brain meninx	1	2	8			F82	68 FANTOM
Caudate nucleus	3	3	13			F60, F76	68 FANTOM
Cerebellum	-	1	11			F60, F76	68 FANTOM
Diencephalon	0.7	4	12			M26(29)	68 FANTOM
Dorsal thalamus	1	4	11			F60, F76	68 FANTOM
Dura mater	2	4	13			F59	68 FANTOM
Globus pallidus	2	5	11			F60, F76	68 FANTOM
Hippocampal formation	2	4	10			F60, F76	68 FANTOM
Locus ceruleus	1	4	9			F60, F76	68 FANTOM
Medulla oblongata	0.9	5	10			F60, F76	68 FANTOM
Middle frontal gyrus	1	3	7			F60, F76	68 FANTOM
Middle temporal gyrus	2	3	11			F60, F76	68 FANTOM
Occipital cortex	2	4	10			F60, F76	68 FANTOM
Occipital lobe	3	3	8			M27	68 FANTOM
Olfactory apparatus	1	3	8			F87	68 FANTOM
Parietal lobe	0.9	3	9			F60, F76	68 FANTOM
Pineal body	1	3	11			F60, F76	68 FANTOM
Pituitary gland	0.9	3	14			F60, F76	68 FANTOM
Putamen	3	4	12			F60	68 FANTOM
Spinal cord	3	3	13			F60, F76	68 FANTOM
Substantia nigra	0.5	1	8			F60, F76	68 FANTOM
Brain	5	2	2	10	1,322	F77	ILLUMINA
	10	2	6	9	894	M66	ENCODE
Cerebellum	20	5	7	23	1,484	F, M	KAESSMANN
Frontal lobe	17	5	5	15	2,332	F, M	KAESSMANN
Prefrontal cortex	10	3	5	16	1,891	M	KAESSMANN
Temporal lobe	20	4	4	12	2,975	M	KAESSMANN

Expression is shown as the number of transcripts of a particular gene per million molecules in the RNA-seq sample. Four different studies have been considered. F: female; M: male; age in years.
