# Supplementary material for: Brain pyrimidine nucleotide synthesis and Alzheimer disease
Source: Aging (Albany NY). 2019 Sep 27;11(19):8433–62. doi: 10.18632/aging.102328 (PMC6814620; doi:10.18632/aging.102328)
Supplement: Supplementary Table 2 [file aging-11-102328-s001.docx]

Supplemental Table 2: Summary of cases used for mRNA expression studies. CA1: hippocampal cornus ammon 1; DG: dentate gyrus; EC: entorhinal cortex; LC: locus ceruleus. CHPC: Clinical and HistoPathological Controls. RIN: RNA integrity number. F: female; M: male. PMD: post-mortem delay. *DHODH* and *UCK2* mRNA levels have been normalized to 10^3^ *GAPDH* mRNAs. The CHPC *MT-CO1* / *18S* ratio mean values have been considered 100 %.
